# Satisfaction with life, affects, and subjective happiness in military personnel

**DOI:** 10.1186/s41155-025-00343-4

**Published:** 2025-04-01

**Authors:** Eva Urbón, Carmen Chafer, Carlos Salavera

**Affiliations:** 1 Investigation Research Group OPIICS, Universidad de Zaragoza, Saragossa, Spain; 2 Cátedra TEA Ediciones Universidad de Zaragoza, Saragossa, Spain

**Keywords:** Satisfaction with life, Affects, Happiness, Military personnel

## Abstract

**Background:**

Satisfaction with life, affects, and subjective happiness in military personnel are significantly correlated, as the experience of positive emotions, such as pride and camaraderie, often improves both satisfaction with life and the perception of happiness. On the other hand, negative effects, such as stress and anxiety, derived from deployment in high-risk environments, can bring down these indicators of well-being. Adaptability and resilience play a crucial role in moderating these affects, allowing military personnel to keep a good degree of emotional balance.

**Objective:**

The objective of this study was to measure satisfaction with life, subjective happiness, and affects on Spanish military personnel. The initial hypothesis was that these variables are correlated and that affects can be used to predict satisfaction with life and subjective happiness.

**Methods:**

This study examined the mutual relationships between satisfaction with life, affects, and subjective happiness in a sample of 416 Spanish military personnel (336 men 80.67% and 80 women 19.23%), with an average age of 35.56 years, using the satisfaction with life, affects, and subjective happiness scales.

**Results:**

The results indicate that all the variables analyzed are correlated and together constitute subjective well-being. Network analysis indicates that the axial factor in this relationship is subjective happiness. It was found that both positive and negative affects can be used to predict satisfaction with life and subjective happiness.

**Conclusion:**

This paper emphasizes the need to conduct more studies with military personnel, who are usually exposed to high levels of professional stress, and that these consider a wider range of variables. Understanding these dynamics is essential to design measures that promote mental health and holistic well-being in this population group. This study is the first empirical approach to models that argue for the mutual interaction of these constructs in psychological well-being, also emphasizing the need to work with military personnel in this regard, not only during their training but throughout their military career.

## Background

Military personnel, deployed in international conflict zones for long periods of time, are often exposed to high levels of stress (fires, floods, response to natural disasters…), and these conditions can affect their mental health in different ways (anxiety, post-traumatic stress, pressure disorders…). Much research has gone into studying mental health, but the mental health of military personnel has been paid little specific research attention. In addition, what research has been carried out is based on self-reporting methods, which can be burdened by the reluctance of the subjects to respond, owing to the stigma associated with seeking help among people whose job is to help others (Warner et al., 2011). Measuring the subjective well-being of military personnel can reveal potential differences between the civilian population and the elements of daily life involved in their search for well-being (Stoltenberg et al., 2024).

Unlike civilians, military personnel face extreme physical and psychological demands, even putting their lives at risk if their duty so requires. Therefore, military personnel must face physical and psychological hazards that other professionals do not endure, and this can affect their physical and psychological health (Bryan et al., 2024). For this reason, they are a particularly interesting subject of research concerning the factors involved in subjective well-being, such as satisfaction with life, affects, and subjective happiness.

It is important to keep in mind that finding happiness or feeling good about one’s life is a life goal and a priority for most people (Diener & Lucas, 2000; Oishi et al., 2024). Society values these two goals, which translate into quality of life and functional individuals, over economic wealth and professional success (Lyubomirsky, 2013; Tucker et al., 2006). However, by its very nature, military personnel often put their professional duties before their personal well-being. Psychology should investigate what factors and processes promote subjective happiness, well-being, and quality of life from a scientific perspective, beyond the human condition and ontological states (Cuadra & Florenzano, 2003), even more so in population groups such as the one addressed in this study, which are oriented towards pursuing constructive aims in conditions of adversity, which can, to an extent, compensate for the difficult circumstances in which they operate. As such, the research priorities must be based on their own discipline-based criteria, context, and values (Hinz et al., 2018; López-Ortega et al., 2016).

The search for subjective well-being is an important field of psychological research that concerns both the social sciences and the health sciences and focuses on the assessment that individuals make of their own lives (Ruggeri et al., 2020; Torres et al., 2014). It has been suggested that satisfaction with life and subjective well-being are not the same thing, with the argument that satisfaction with life is one of the components of subjective well-being, essentially the assessment that individuals make of their daily lives (Pavot & Diener, 1993; Vitterso et al., 2005). This is a basic personal trait, particularly among military personnel. We need to understand what makes military personnel feel good about themselves according to their own values and standards, which are often far removed from those of the general population (Diener et al., 2003; Kring et al., 2024). That is, our approach should not be based on externally imposed standards, but on a subjective assessment of quality of life within the subject's own ideal, in this instance, the subjective perception of being closer to meeting a life goal in a military context (Esnaola et al., 2019).

## Subjective well-being

### Satisfaction with life

According to Diener (1985), satisfaction with life is a subjective assessment. The cognitive component of satisfaction with life is a global comparison of goals achieved with challenges and expectations (Ott, 2020), and it is a component of the broader notion of subjective well-being. The cognitive-evaluative aspect (Diener, 1984; Wu et al., 2024) is key, expressing the individual's assessment of their quality of life against their own criteria and personal experience. Therefore, cognitive and emotional elements should be analyzed separately, distinguishing between current or short-term emotional state and satisfaction with life, which is a more abstract, cognitive, and subjective continuum (Vázquez et al., 2013).

Diener and his collaborators conducted a cross-cultural study that covered more than 39 countries. A key conclusion of this study was that satisfaction with life (a need shared by all human beings), depended largely on the individualistic/collectivist nature of the specific social context, as it is in itself a social concept. In any case, the study of satisfaction with life is interesting insofar as it reveals important information about individuals or groups, but it is also important in terms of decision-making in many fields of public policy, including public transport, healthcare, education, etc.

The Satisfaction with Life Scale (Diener et al., 1985) is used to scientifically study this construct, while other psychometric tools are used to measure emotions. The scale was designed to measure satisfaction with life, which, as noted, is the cognitive component of subjective well-being (Lewis et al., 1999). With this scale, the subject makes a global assessment of satisfaction with life from a cognitive perspective, without taking into account specific variables (e.g., health, vitality) (Brailovskaia et al., 2024). This self-report instrument was designed to measure satisfaction with life from its affective and cognitive perspectives, referring to the feelings of pleasure and displeasure that reflect the subjective well-being of the individual. Well-being is the result of maximizing positive feelings and minimizing negative ones, both of which are also indicative of the subject’s emotional competence (Aishvarya et al., 2014).

### Subjective happiness

Happiness is one of the constructs of Positive Psychology, which examines the beneficial impact of positive variables on human health (Karakasidou et al., 2016). According to Iani (2014), there are two approaches to studying happiness: one focuses on psychological well-being, that is, human potential; the other focuses on subjective well-being, that is, the subjective evaluation of everyday events. These two concepts of happiness are generally referred to as eudemonic and hedonic well-being, respectively. A happy person has a positive temperament and the ability to see the positive side of everyday life; they do not dwell excessively on things; they have a social network that they trust; and they can mobilize tools and personal resources to face challenges (Hernández & Landero, 2014). However, there is no widespread agreement on the definition of happiness, whether it refers to individuals, groups, or institutions (Karakasidou et al., 2016). However, there is no doubt about its physical and psychological benefits and its role in strengthening individual coping mechanisms (Rodowicz et al., 2020). In any case, happy and unhappy individuals differ in their responses to everyday life; happy people perceive, interpret, and think about events in a more positive light (Kraft & Kraft, 2023; Lyubomirsky & Tucker, 1998).

Although the concept of happiness can change depending on age, country, and culture, and even on different stages of an individual's life cycle (Lyubomirsky, 2005), positive psychology theory always stresses the importance of our psychological strengths and satisfaction with life as a shield against psychopathologies (Seligman, 2003). For this reason, studies such as that of Kubzansky et al. (2023) emphasize the role of optimism, satisfaction with life, positive affects, and life purpose as mechanisms to improve vitality and reduce the danger of harmful behaviors and premature death.

In an imperfect world, in which our lives are imperfect too, it is to be expected that happiness can never be total, depending more on subjective impressions than on objective facts. As such, most of the population sees happiness as a difficult goal; changing our personal circumstances (earning more money, changing marital status, graduating, changing jobs, etc.) does not guarantee greater happiness (Layous & Lyubomirsky, 2014). It is its subjective nature that makes happiness so difficult to define operationally in a way that frames emotional states and enduring cognitions, as well as to measure because science needs to work with data, not art or philosophy. The complexity of the concept is also reflected in the gray area generated by the overlapping use of the term in scientific and everyday language (Babincak, 2018). This is why it is so important to make happiness a measurable construct, even if it can only be measured subjectively by the individual (Chai, 2023).

### Affects

Following the studies of Bradburn and Caplovitz (1965) and Bradburn (1969), which examined the two dimensions of the so-called Psychology of Well-Being and positive and negative affects, the study of affects has been both fruitful and controversial, giving rise to two models: one describes affects as a group of dimensions, which are different but systematically related; pleasure and arousal are considered distinct (Russell, 1980) and their relationship is problematic (Russell & Carroll, 1999). The other approach considers two factors: positive affects and negative affects (Watson & Tellegen, 1985), in line with the general structure of affects put forth by Diener et al. (1985), which confirms the coexistence of positive and negative factors, as defined by Joshanloo (2023).

In 1988, David Watson, Lee Anna Clark, and Auke Tellegen developed the affects scale, which includes both positive and negative affects. Affects are understood here as a state of mind, so the scale measures this more than emotional factors.

To do this, they established a two-dimensional orthogonal model, with two independent variables. Positive affects translate into an active, alert and enthusiastic attitude, and negative affects into sadness or idleness. High scores in the negative affect dimension reflect distress, while low scores represent serenity and calmness, suggesting that negative affects can be subcategorized by degree and also lead to more complex affective structures (Yarrington & Craske, 2024).

### Satisfaction with life, affects, and happiness

The relationship between affects, satisfaction with life, and subjective happiness has been extensively researched in psychology, which has presented evidence that strongly suggests that these constructs are interrelated in very significant ways (Berscheid & Regan, 2019; Diener et al., 2018a, 2018b; Kashdan & Rottenberg, 2019; Tov & Tkach, 2019). In military personnel, adaptability and resilience play a crucial role in moderating these affects, allowing individuals to keep a good degree of emotional balance, which is key when undergoing experiences that generate positive emotions, as well as pride and camaraderie, which increase their satisfaction with life and their perception of happiness. On the other hand, negative emotions, such as stress and anxiety, can result from high-risk deployments or environments. Recent studies have shown that positive affects, such as joy and enthusiasm, are strongly associated with higher levels of satisfaction with life and subjective happiness (Kushlev et al., 2020a, 2020b). On the other hand, negative affects, such as sadness or stress, tend to decrease satisfaction with life and subjective happiness, negatively affecting overall well-being (Ng & Tov, 2019). In this way, different studies have found that people who experience positive affects frequently tend to assess their lives more favorably, which in turn increases their subjective happiness (Busseri, 2018; Huta & Waterman, 2020). In addition, the ability to regulate emotions plays a crucial role in this relationship, as it facilitates the management of negative affects and promotes the experience of positive emotions (Gross, 2020). The theory of positive–negative affects suggests that the predominance of positive affects predicts higher levels of subjective happiness (Tov, 2018), given that subjective happiness is nourished by both positive affects and satisfaction with life, which makes it a consolidated global indicator of psychological well-being (Lyubomirsky & Layous, 2021). These affects can directly influence the individual’s global assessments of satisfaction with life, as they are interpreted as indicators of a fulfilling life (Tov et al., 2019).

On the other hand, the relationship between affects and happiness is also explained by the influence that these have on sustained emotional states, generating a positive cycle that reinforces general well-being (Kashdan et al., 2018). In this way, positive affects not only reflect but can also predict, both happiness and satisfaction with life, underscoring the importance of fostering positive emotional experiences to improve psychological well-being.

The aim of this study is to measure a set of psychological variables (satisfaction with life, subjective happiness, and affects) in Spanish military personnel. The initial hypotheses of the study are as follows: (1) satisfaction with life, subjective happiness, and affects are correlated in this population; (2) affects, both positive and negative, predict satisfaction with life and subjective happiness; and (3) subjects that yield high scores in satisfaction with life will also yield high scores in subjective happiness and show high positive affects, while people with low scores in one of these constructs will also show low scores in the other two.

## Methodology

### Participants and protocol

Prior to the study, military barracks were contacted by telephone to request their cooperation. The target barracks, in the region of Aragon (Spain), were selected by simple random sampling. The objectives of the study were explained to the participants and the importance of responding to all items was emphasized. Participants had twenty minutes to fill out the questionnaires and sign the informed consent form. Participants were informed that the responses would be treated anonymously and confidentially.

The study comprised 416 participants (336 men, 80.67%, and 80 women, 19.23%), with an average age of 35.56 years; the sample is representative of the Spanish armed forces (Spanish Ministry of Defence, 2023). Incomplete and incorrectly completed questionnaires (*n* = 8) were excluded. All participants were volunteers and signed an informed consent form, following the ethical guidelines set out in the Declaration of Helsinki and standard ethical principles for research involving human subjects. To check that the questionnaires were understood correctly, a small sample of participants (*n* = 25) was selected prior to the survey. The final survey was conducted in the presence of the researchers, and questionnaires were collected individually in order of completion to check for possible errors or unanswered items. In the definition of Ato and Vallejo (2015), this study is an ex facto prospective survey with a simple descriptive design. The results were processed anonymously. The representativeness of the sample was calculated using a sampling formula (Rudolph et al., 2023), which yielded a 99% level of confidence, suggesting that the sample is representative (Table 1).

**Table 1:** Descriptive statistics of the sample

		*N*	%
Sex	Men	336	80.77
	Women	80	19.23
Age	Under 20 years	9	2.16
	20 to 30 years	202	48.56
	31 to 40 years	91	21.88
	Over 40 years	114	27.40
Educational level	Secondary school	45	10.82
	Primary school dropout	16	3.85
	Primary school	102	24.52
	Trade training dropout	10	2.40
	Trade training	98	23.56
	University drop out	35	8.41
	University graduate	35	8.41
	Doctorate	1	0.24
	Basic training	40	9.62
	Officer training	34	8.17
Marital status	Single	141	33.89
	Married	124	29.81
	In partnership	130	31.25
	Separated or divorced	19	4.57
	Widower/widow	2	0.48
Years in the army	1 year or less	75	18.03
	2 to 5 years	108	25.96
	6 to 10 years	43	10.34
	11 to 20 years	90	21.63
	Over 20 years	100	24.04

### Instruments

The Satisfaction with Life Scale (Diener et al., 1985) was developed to measure subjective well-being. The Spanish version of the scale was used in this study (Atienza et al., 2000). Answering the scale takes no more than a minute. It is divided into five items on a seven-point Likert scale with responses ranging from 1 (strongly disagree) to 7 (strongly agree). As such, total scores range from 5 to 35; high scores suggest greater satisfaction with life. The scale yielded a Cronbach’s alpha value of 0.87.

The Subjective Happiness Scale (Lyubomirsky & Lepper, 1999) presents a molar measure of well-being as a global psychological phenomenon. The scale goes beyond the mere aggregation of positive and negative emotions and related cognitive states. Therefore, the scale measures happiness from the point of view of the interviewee, with the understanding that, although different variables can be used to describe happiness, each interviewee will have his or her own perspective of what happiness is, and whether he or she is happy. It is divided into four items with answers arranged on a Likert scale; scores are added together and divided by the total number of items. In this study, the Spanish version of this scale was used (Extremera & Fernández-Berrocal, 2014) and the scale yielded a high value of internal consistency (Cronbach's alpha = 0.84; Ω = 0.83).

The Positive and Negative Affects Scale (Watson et al., 1988) is divided into 20 items, 10 for positive affects and 10 for negative affects, expressed on a Likert scale. The Spanish version of this scale was used in this study (Sandin et al., 1999). The answers must refer to the present tense and range from 0 (absence of the emotion) to 5 (frequent presence of the emotion). The questionnaire yielded a Cronbach’s alpha value of 0.88 for positive affects and 0.86 for negative affects.

In addition to these, participants completed an ad hoc questionnaire to ensure that they all presented comparable psychological states and to facilitate the tabulation and comparison of responses (Berinsky, 2017). In addition, socio-demographic data (sex, age, marital status, educational level, and seniority in the army) were also collected.

### Data analysis

Data was collected in September and October 2023. The statistical analysis was performed with SPSS 26.0 software. Following standard normality tests (normality, independence, homoscedasticity, and linearity), parametric techniques were chosen to outline the sociodemographic profile of the data and the various variables studied. Descriptive statistics were used. Correlations between satisfaction with life, subjective happiness, and affects (positive and negative) were calculated before being processed and analyzed with SPSS 26.0 (IBM, Armonk, NY, USA). Multiple regression by stages was used to estimate the predictive value of affects, both positive and negative, on subjective happiness and satisfaction with life. For all operations, a significance level of *p* 0.05 was adopted, with a level of confidence of 95%. The analysis used direct scores, as recommended by the questionnaire use manuals. Average clusters were established to detect clusters and distribute participants into groups based on satisfaction with life, affects, and subjective happiness scores. In addition, the JASP software, v. 0.10.2 (JASP Team, 2019) and the Fruchterman-Reingold algorithm (Fruchterman & Reingold, 1991) were used to perform undirected and weighted network analyses, to assess the structural dynamics between reactors and to focus on the specific contribution of each indicator (Blasco-Belled et al., 2024). An item-level analysis was carried out to analyze whether the network is grouped into different communities, which would help to understand how items make up separate dimensions and the importance of each dimension within the network structure (Blasco-Belled, 2022). The graphs illustrate the nodes, which represent the indicators of the variables, and the edges (lines), which represent the internodal relationships (Hevey, 2018; Schmittmann et al., 2013). The parameters considered are the Ebiclasso estimator (minus absolute contraction and selection graph operation) (Friedman et al., 2008), which allows a stable network based on regularized and easy-to-interpret partial correlations, with an estimation index of 0.50 (Foygel & Drton, 2010), which is recommended to generate more stable and parsimonious networks. Similarly, centrality indices were calculated to estimate the interconnectedness of the grid structure (Opsahl et al., 2019). The most central indicators have a greater influence on the rest of the variables in the network (Epskamp & Fried, 2018). These indicators reflect the degree of connectivity (centrality and intermediation), the proximity between all the variables of the network (centrality of proximity), and the frequency of connections of each node with all possible connections (centrality of force) (Blanco et al., 2019). These are the most important and widely used measures of centrality in network analysis (McNally, 2021).

## Results

First, the results of the variables studied (satisfaction with life, positive and negative affects, and subjective happiness) were analyzed, including the calculation of means and standard deviations (Table 2).

**Table 2:** Results (average and standard deviation) of the variables under study

	*x*	s.d
Satisfaction with life	25.34	6.35
Positive affects	38.38	6.32
Negative affects	19.60	7.76
Happiness	21.46	4.21

To evaluate the relationships between the variables, following the first hypothesis, the correlations between satisfaction with life, affects (positive and negative), and subjective happiness were calculated (Table 3). The results showed that satisfaction with life has a strong positive correlation with positive affects (*r* = 0.52**) and happiness (*r* = 0.65**), and a strong negative correlation with negative affects (*r* = − 0.50**), indicating that the higher the satisfaction with life, the less prevalent the negative affects are. On the other hand, happiness and positive affects were also positively correlated (*r* = 0.52**), and negative affects were negatively correlated with subjective happiness (*r* = — 0.57**) and positive affects (*r* = − 0.39**).

**Table 3:** Correlations between variables

		1	2	3	4
1	Satisfaction life	–			
2	Positive affect	.52**	–		
3	Negative affect	− .50**	− .39**	–	
4	Happiness	.65**	.59**	− 0.57**	–

^**^p < .001

**Fig. 1:**
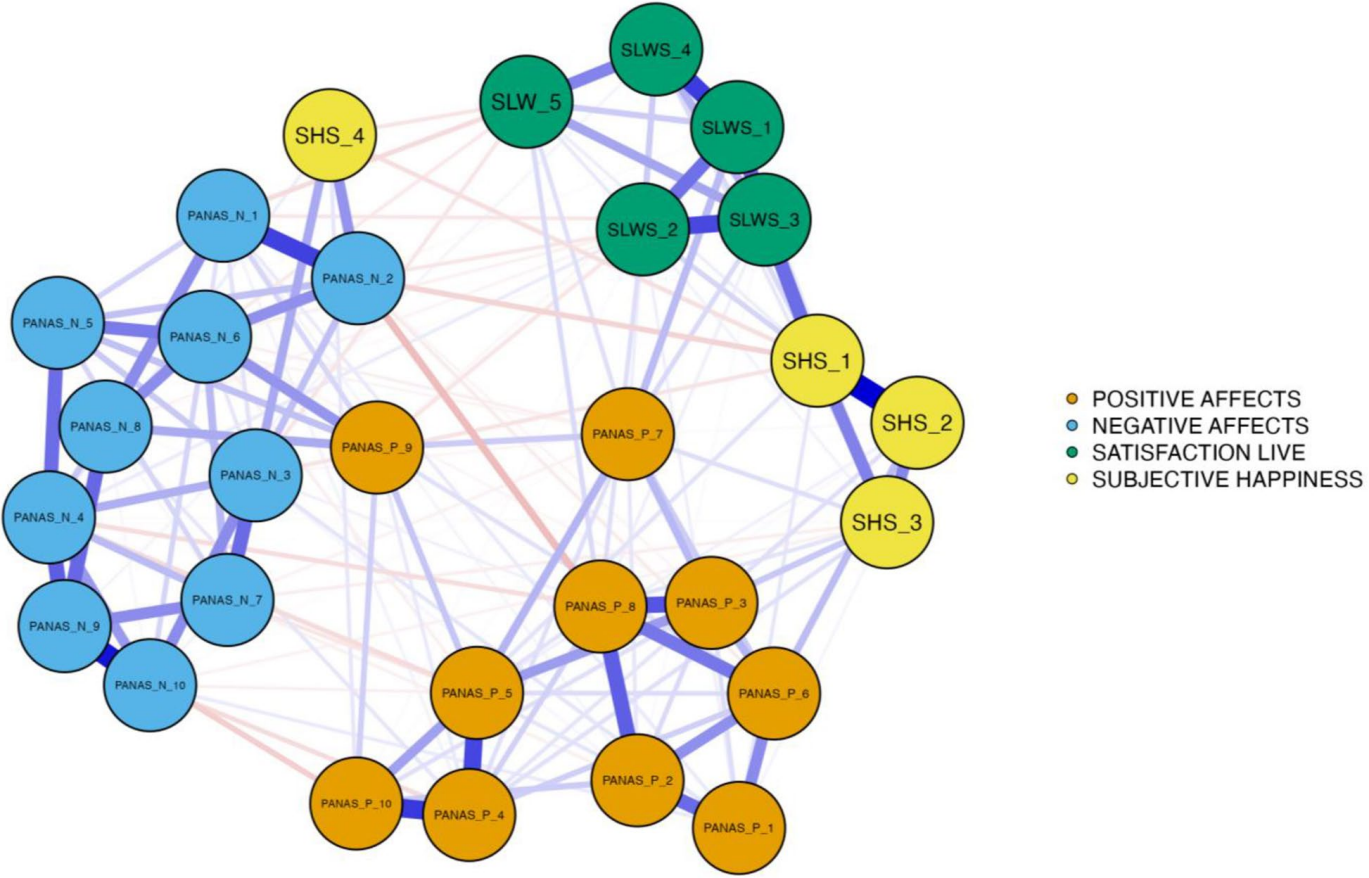
Network analysis of the constructs under analysis

In relation to the first hypothesis, the constructs were subjected to network analysis, using the items as units of analysis (Fig. 1), in order to explore the relationships between satisfaction with life, subjective happiness, and positive and negative affects. The blue edges or lines represent positive correlations between the nodes, indicating that an increase in the score of one of the elements leads to an increase in the score of the connected elements. On the other hand, red lines signal negative correlations between nodes. As expected, all items within each scale were positively correlated with each other. However, some items showed unexpected correlations outside of their own scales. For instance, the SHS 4 item (Some people are generally not very happy…) showed strong correlations with the negative affect scale (PANAS-N). Similarly, the item PANAS 9 (Attentive) of the Positive Affects Scale (PANAS-P) was closer to the items on the Negative Affects Scale than to those on its own scale. Regarding negative correlations, a significant number of relationships between items were observed in the two affect scales. Specifically, negative correlations were found between the item PANAS-P 8 (Determined) and PANAS-N 2 (Upset) with a partial score of *r* = − 0.11, *p* < 0.01; items PANAS-P 10 (Inspired) and PANAS-N 10 (Afraid) with *r* = − 0.08, *p* < 0.01; and items PANAS-P 7 (Inspired) and PANAS-N 5 (Proud) with *r* = − 0.04, *p* < 0.01. These results suggest that, although weakly, a higher presence of one type of affect is associated with a lower presence of the other. This finding is particularly interesting, given that, according to the theoretical structure of the model, positive and negative affects are orthogonal dimensions, implying that they should be independent of each other.

Following the second hypothesis, multiple hierarchical regressions were carried out to establish the predictive value of affects (positive and negative) on satisfaction with life (Table 4) and subjective happiness (Table 5), using the scores of positive and negative affects as predictors and subjective happiness as a criterion variable.

**Table 4:** Affects as predictors of satisfaction with life

		*B*	e.t	*R*^*2*^	*t*	Sig
1	Constant	25.34	0.31		81.43	< .001
2	Constant	5.15	1.63	.52	3.15	< .001
	Positive affect	0.53	0.04		12.52	< .001
3	Constant	15.86	2.02	.61	7.86	< .001
	Positive affect	0.39	0.04		9.17	< .001
	Negative affect	− 0.28	0.03		− 8.08	< .001

**Table 5:** Affects as a predictor of happiness

		*B*	e.t	*R*^*2*^	*t*	Sig
1	Constant	21.46	0.21		104.01	< .001
2	Constant	6.43	1.03	.59	6.25	< .001
	Positive affect	0.39	0.03		14.82	< .001
3	Constant	14.60	1.22	.69	11.95	< .001
	Positive affect	0.29	0.03		11.18	< .001
	Negative affect	− 0.21	0.02		− 10.19	< .001

Table 4 presents the steps followed to introduce explanatory variables with a significant predictive value in relation to satisfaction with life into the model. Positive affects produced significant positive affects (*B* = 0.39, *p* < 0.001) and negative affects produced significant negative affects (*B* = − 0.28, *p* < 0.001); the result of the Durbin-Watson test suggests that the errors are independent. For the regression model used, these predictor variables explain 61% of the variance of the dependent variable (*R*^2^ = 0.61).

Table 5 presents the steps taken to introduce explanatory variables with a significant predictive value in relation to subjective happiness into the models. In this case, the values yielded by the predictor variables were somewhat lower than in the previous model. Positive affects produced significant positive affects (*B* = 0.29, *p* < 0.001) and significant negative affects (*B* = − 0.21, *p* < 0.001); the result of the Durbin-Watson test suggests that the errors are independent. For the regression model used, these predictor variables explain 69% of the variance of the dependent variable (*R*^2^ = 0.69).

In addition, cluster analyses were undertaken (Table 6) to divide participants into groups according to their satisfaction with life, affects, and subjective happiness. Three main groups were identified: (1) 28 participants (6.73%) with below-average scores in terms of satisfaction with life, positive affect, and subjective happiness, and well above average in terms of negative affects; (2) 261 participants (63.19%) with above-average scores in terms of satisfaction with life, subjective happiness and positive affects, and below-average scores in terms of negative affects; and (3) 127 participants (30.75%) with near-average scores in terms of satisfaction with life, subjective happiness, and positive affects and somewhat above average negative affects. In all groups, the differences were significant (*p* < 0.001).

**Table 6:** Cluster analysis on satisfaction with life, affects, and subjective happiness

	G1	G2	G3	*x*	*F*	Sig
Satisfaction with life	12.82	28.37	21.87	25.34	204.17	.000
Positive affects	25.04	40.36	37.26	38.38	122.25	.000
Negative affects	34.71	15.19	25.32	19.60	344.42	.000
Subjective happiness	12.21	23.34	19.64	21.46	214.13	.000
*N* (%)	28 (6.73%)	261 (63.19%)	127 (30.75%)			

**Fig. 2:**
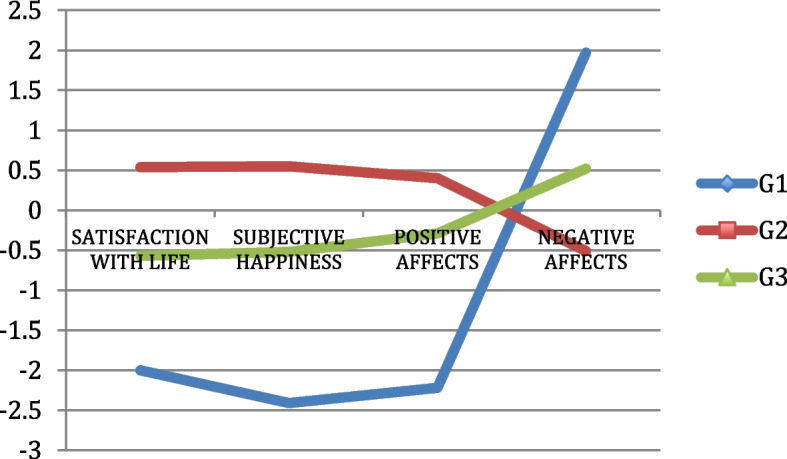
Groups of participants grouped by satisfaction with life, affects, and subjective happiness

Figure 2 illustrates cluster analysis. *Z*-scores were used to facilitate the visualization of the graph, outlining the profile of the participants in terms of satisfaction with life, affects, and subjective happiness, comparing individual and average scores. As shown in the figure, regardless of sociometric factors, participants in group 1 yielded scores that were well below average in terms of satisfaction with life, subjective happiness and positive affects, and above average in negative affects. On the other hand, the scores yielded by groups 2 and 3 were much closer to mean values.

## Discussion

Academic interest in well-being has increased in recent decades. Well-being is not only defined by the absence of disease, but also includes other factors, such as a healthy life, both physical and psychological, in the workplace and in our everyday social circles. In recent years, the number of sick leaves has increased, especially after the pandemic. Demands and professional stress converge with personal and professional exhaustion. While most studies focus on the impact of negative factors, this study takes a more positive outlook, focusing on well-being.

Since this study concerns military personnel, it is not surprising that the sample includes four times as many men as women. Half of the interviewees were under 30 years of age and three out of four were under 40 years of age; 60% were in a stable relationship (married or not) and one in six was a university graduate. About half had been in the military for 10 years or more.

The correlation analysis found partial correlations between the different variables used to measure psychological well-being; subjective happiness, satisfaction with life, and positive affects were found to be positively correlated. All of these were negatively correlated with negative affects, indicating that higher levels of satisfaction with life, happiness, and positive affects help to fend off negative affects. This suggests that sound emotional management can greatly contribute to individual well-being, in line with what has been suggested in previous studies (Kring et al., 2024; Sanín-Posada et al., 2019).

This is the first time that network analysis has been used to study the mutual structural relationships between happiness, satisfaction with life, and positive and negative affects. In addition, this analysis was carried out at the item level, which gives it greater depth (Blasco-Belled, 2022). The systemic interactions of network analysis point to a close relationship between happiness and satisfaction with life, suggesting that subjective happiness and satisfaction with life may be the key link to emotional well-being, a conclusion shared widely (Diener et al., 2013; Lyubomirsky & Lepper, 1999; Seligman, 2003) that has been reinforced by the results of recent studies (Oishi et al., 2024; Trachik et al., 2023), including ours. Our analysis of these results follows Diener and Lucas (2000), who analyzed what social factors can contribute to improving individual subjective well-being, formulating the Evaluation Theory. According to this theory, attention is selective, so subjective well-being will depend on how the individual evaluates events based on their own needs, goals, and culture. In addition, cognitive and emotional self-assessment can actively promote feelings of content, fulfillment, and happiness, although external and cultural variables also play a role. This shows that subjective well-being is a global and stable phenomenon and not a hasty judgment based on circumstance. In this section, the relationship of item 4 in the subjective happiness scale with negative affects is highlighted, as already pointed out by earlier studies (Barraza, 2020; O'Connor et al., 2015; Morillo, 2013), which deserves a more in-depth analysis in the future. This item, although part of the subjective happiness scale, showed greater correlations with negative affects than with the elements in its own scale. The correlations observed between items of the two scales of affect are also worth pointing out. This scale is theoretically orthogonal (Watson & Tellegen, 1985), but in this study item 9 in the positive scale was more closely related to the scale of negative affects than to the items in its own scale, even if the negative correlations found were weak (Crawford & Henry, 2019), suggesting the need to further investigate this phenomenon in future studies.

The relationship between happiness and the rest of the variables is of great interest. Happiness is very important and an important factor in success. Happy people are successful and achieve their goals in all areas of life (health, work, studies) and this happens because happy people change circumstances in their favor, investing their personal resources (confidence, optimism, prosocial behavior, self-efficacy, constructive attitude, creativity, kindness, and coping skills) (Gladstone et al., 2024). In addition, happiness has a direct effect on physical and psychological health; happier people feel healthier and better prepared to face difficult situations (Rodowicz et al., 2020).

Regarding affects, in line with previous studies (Robles & Páez, 2003; Watson et al., 1988), the results suggest that positive and negative affects are not the two poles of a continuous spectrum, but two different and relatively independent dimensions. Positive and negative affects are not interrelated, although some authors argue in favor of there being a moderate mutual influence (Crawford & Henry, 2004).

### Limitations of the study

The limitations of this study should be taken into account. Although the sample is statistically representative, it is limited to military personnel from the Aragon region of Spain, so extrapolating the results to other regions or branches of the armed forces (e.g. air force or navy) should only be done with great caution. The sample, although representative of the personnel employed by the Spanish army, was predominantly made up of men (80.67%), which may limit the applicability of the results to female military personnel or mixed-gender groups. In addition, it is a cross-sectional study, which means that it captures data at a single point in time and limits our ability to infer causal relationships or observe changes in variables over time. Another limitation is the use of self-reported measures, which can introduce bias, as participants may underestimate negative emotions or overestimate positive ones owing to social desirability or stigma associated with mental health in military settings. On the other hand, the study focused on satisfaction with life, affects, and subjective happiness, but did not explore other relevant psychological variables (e.g. resilience, coping mechanisms, or trauma) that could provide a more complete understanding of the well-being of military personnel. Finally, the study was conducted over a short period (September and October 2023), so future longitudinal studies could provide a better understanding of how these variables evolve over time, especially in response to stressors such as deployments or promotions, which is why longitudinal studies are recommended for future research on this topic.

### Future prospects

The inclusion of the network model was the most outstanding contribution of the study. The use of this statistical method is not widespread, although it offers better results than other methods by drawing multivariate associations from a systemic perspective, which is eminently suitable for the study of psychological phenomena. It is important to determine the role that well-being plays among military personnel, and it would be desirable to work with these professionals to improve their emotional well-being, reduce their stress, and improve their professional performance and job satisfaction. In addition to dealing with their own personal challenges, military personnel must face complex tasks, often in conflict zones, which can place a heavy burden on their well-being.

## Conclusion

The study found that subjective happiness, satisfaction with life, and positive affects were positively correlated, indicating that sound emotional management contributes to the individual’s well-being. In addition, a relationship between happiness and satisfaction with life was observed, contributing to individual subjective well-being, which depends on how individuals evaluate events based on their own needs, goals, and culture. Finally, the results show that positive and negative affects are not the two poles of a continuous spectrum, but two different and relatively independent dimensions that influence a person's subjective well-being.

Finally, the information provided in this study can be useful at both a scientific and an educational level. On the one hand, the exhaustive analysis of psychological well-being in military personnel, through a battery of self-reports, allows us to better comprehend how to integrate psychological well-being into the daily work of these professionals, which sometimes implies high levels of stress. On the other hand, this exhaustive and detailed analysis is essential for military personnel to know how to better interiorize these psychological well-being variables, encouraging their professional development. In addition, a better theoretical understanding of the variables that affect well-being will allow professionals involved in the training of military personnel to adjust them more effectively to their own goals, professional performance, and the specificities of the military context within which they work.

## Data Availability

The data presented in this study are available upon request from the corresponding author.
